# Supplementary material for: A general patterning approach by manipulating the evolution of two-dimensional liquid foams
Source: Nat Commun. 2017 Jan 30;8:14110. doi: 10.1038/ncomms14110 (PMC5290267; doi:10.1038/ncomms14110)
Supplement: Supplementary Information — Supplementary Figures 1-20, Supplementary Notes 1-3 and Supplementary References [file ncomms14110-s1.pdf]

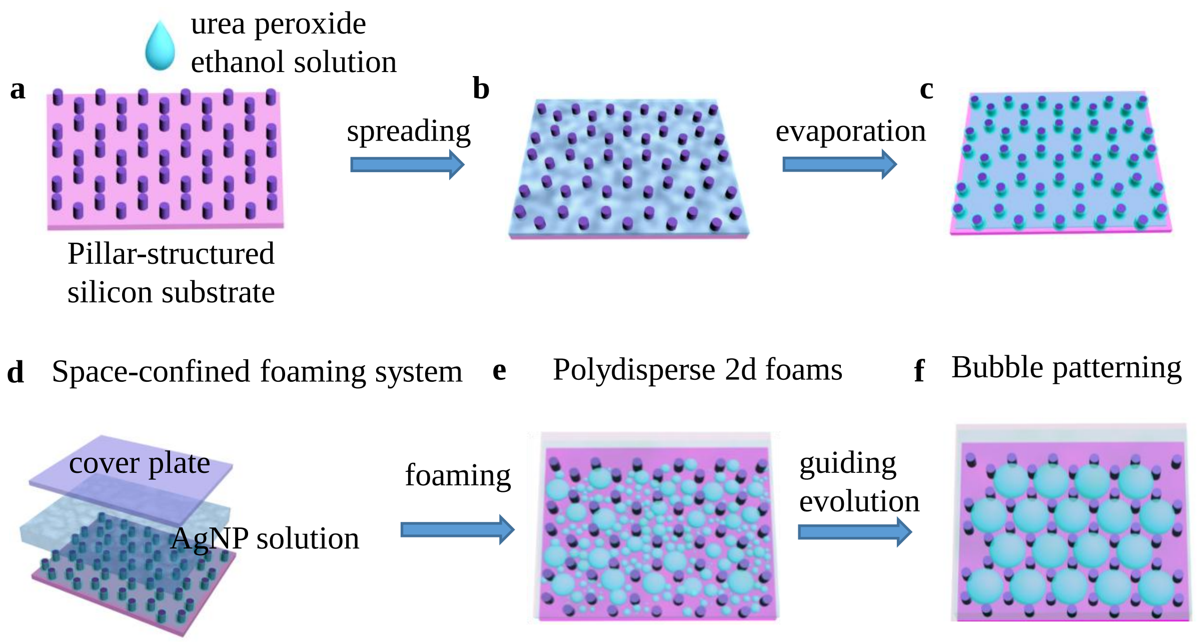

Supplementary Figure 1 │ Schematic illustration of the experimental process. **(a-d)** Preparation of the 2D gas-liquid foams. **(e-f)** Guiding the 2D gas-liquid foams evolving into patterns as desired. **(a-b)** A drop of urea peroxide ethanol solution spread on the pillar-structured silicon substrate because of the hydrophilicity of the substrate. The ethanol solution of urea peroxide was chosen because it was easier to spread on the patterned substrate and uniformly distribute than that of hydrogen peroxide water solution. **(c)** After the solution evaporated with pillars as wetting defects[1], urea peroxide almost uniformly distributed on the substrate. **(d)** The AgNP solution was dropped on the cover plate, and carefully turned around the cover plate, then put it on the as-prepared substrate, forming a sandwiched structure. **(e)** As the decomposition of urea peroxide catalyzed by AgNPs, polydisperse 2d foams were generated. **(f)** With the guidance of pillars on the patterned substrate, 2D gas-liquid foams evolved into the designed patterns.

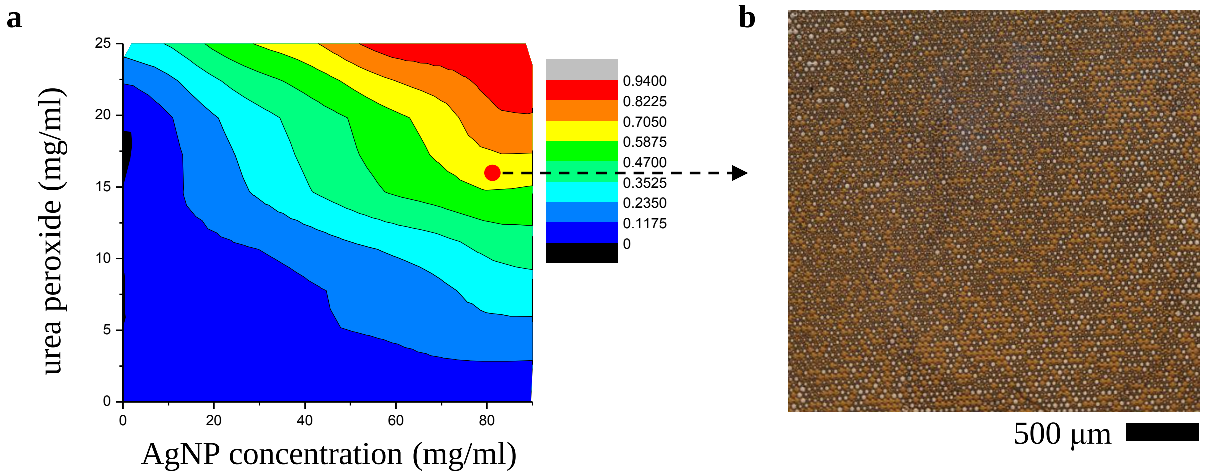

Supplementary Figure 2 │ 2D gas-liquid foams with different gas volume fraction can be prepared by varying the concentration of AgNP solution and urea peroxide ethanol solution. **(a)** Dependence of gas volume fraction in 2D foams on concentrations of AgNPs and urea peroxide. Gas volume fraction was estimated by the ratio of bubbles area with total area from microscope observation. **(b)** Top view of microscope observation of 2D foams with conditions indicated in **a**.

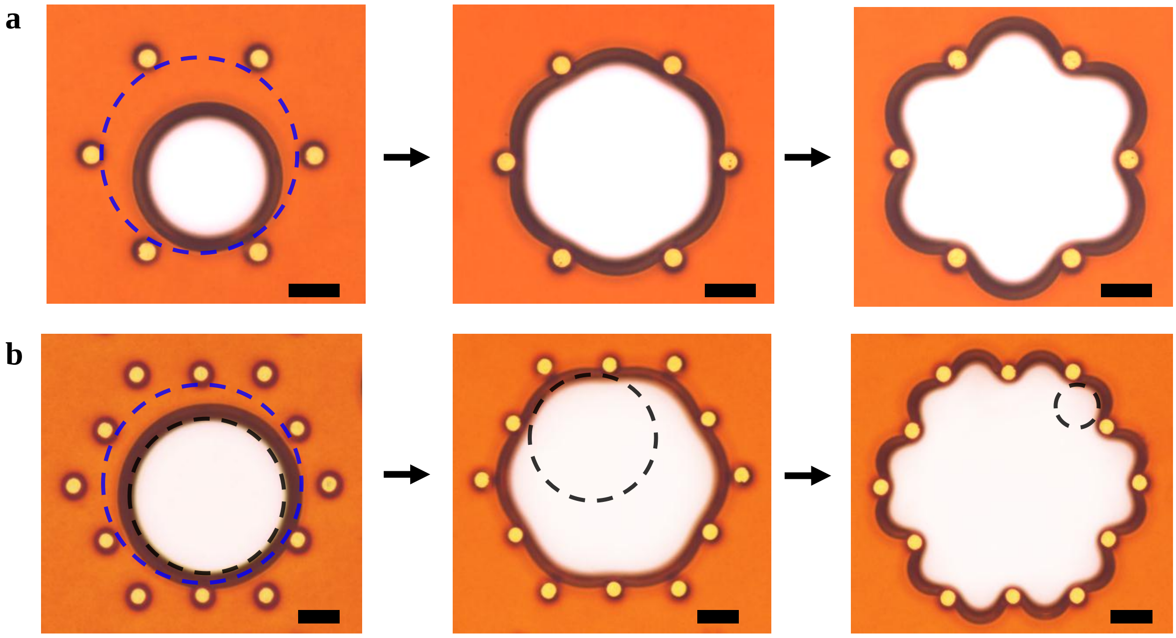

Supplementary Figure 3 │ Microscope observation from a top view of the single bubble grows and deforms in a hexagonal pillar cell with two (**a**) and three (**b**) pillars in each side. The blue dashed circles in **a** and **b** denote the maximum circular size of bubbles growing in the hexagonal cells. The change in radius of the black dashed circles in **b** indicates the change in radius of curvature of the bubble during bubble growing. When a bubble meet pillars, its boundary will deform into several meniscuses. The more pillars in each hexagonal side, the more meniscuses will be formed. In addition, the bubble has a less radius of curvature after interacting with pillars. Scale bars, 50 μm.

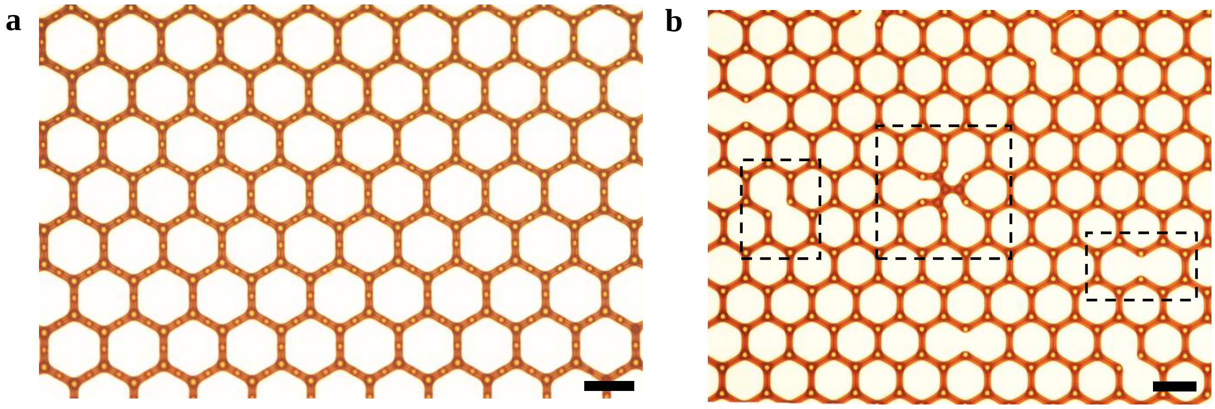

Supplementary Figure 4 │ When the pillar interval increased from 35 μm (**a**) to 60 μm (**b**), some defects formed, as donated by black dotted square in **b**. It shows that the pillar interval should be well designed for preparing perfect hexagonal bubble arrays. Scale bars, 100 μm.

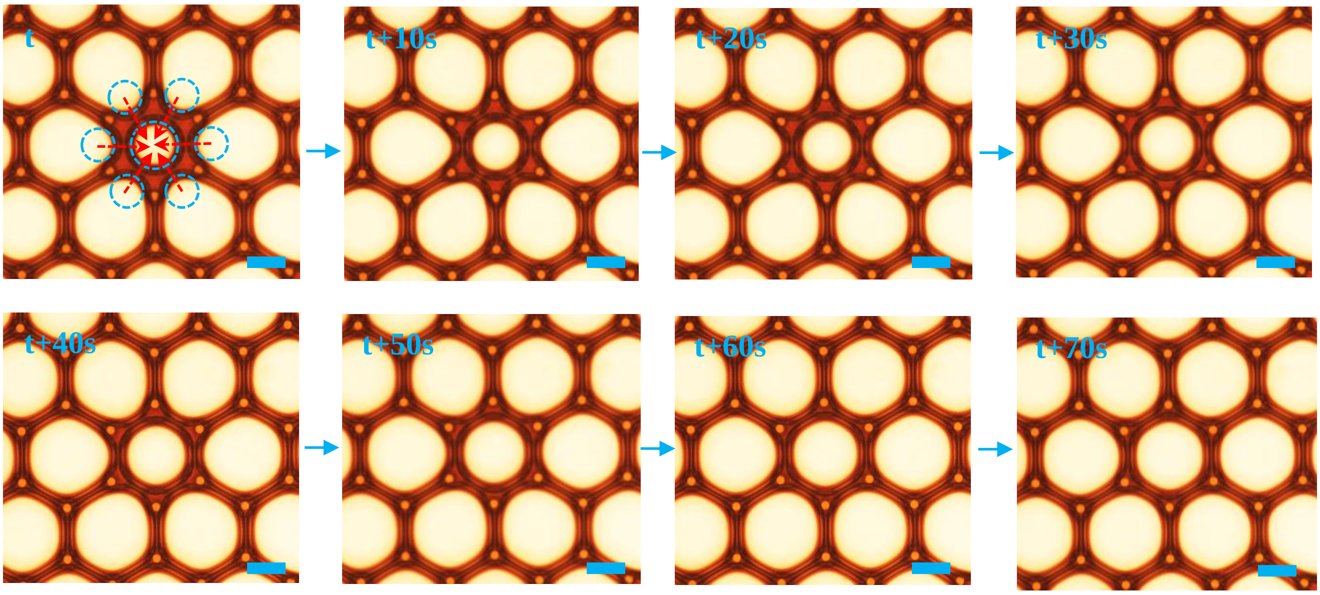

Supplementary Figure 5 | The longer series of images corresponding to Fig. 2a for exhibiting the reverse Ostwald ripening in detail. The red arrows denote the gas transfer direction among the central bubble and the surrounding bubbles by cause of differences in radii of curvature (denoted with blue dashed circle). Scale bars, 50 μm.

a b c

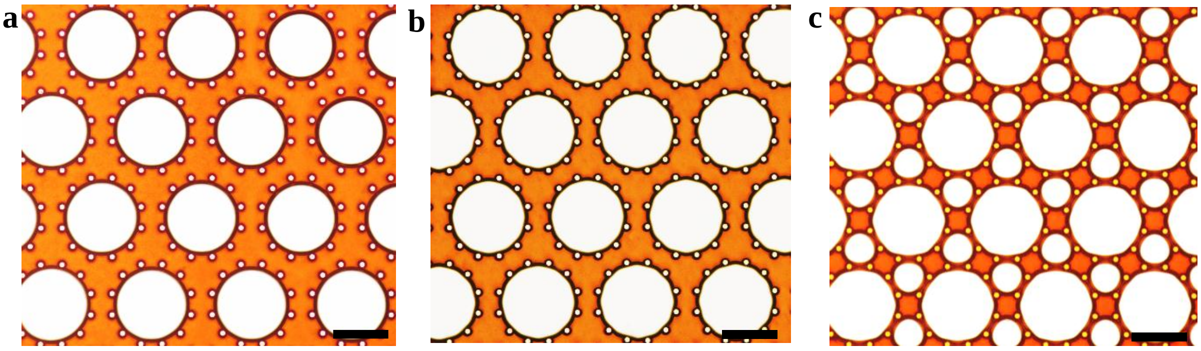

Supplementary Figure 6 │ 2D bubble patterns prepared with the identical pillar-structure silicon substrate by changing the gas volume fraction. (a) When the total gas is not sufficient (gas volume fraction less than 57%), the bubbles in the dodecagonal cells will adsorb all the adjacent bubbles in square and hexagon cells and still maintain circular. **(b)**, A little more gas than that in **a**, the deformed bubble arrays would be obtained. **c**, If the total gas is sufficient (gas volume fraction is about 84%), the bubbles in hexagon can survival because the consumption of the bubbles in the square cells has filled the dodecagonal cells. The radii of curvature for the bubbles in hexagonal and dodecagonal cells are equal, therefore, the bubble pattern is very stable. Scale bars, 100 μm.

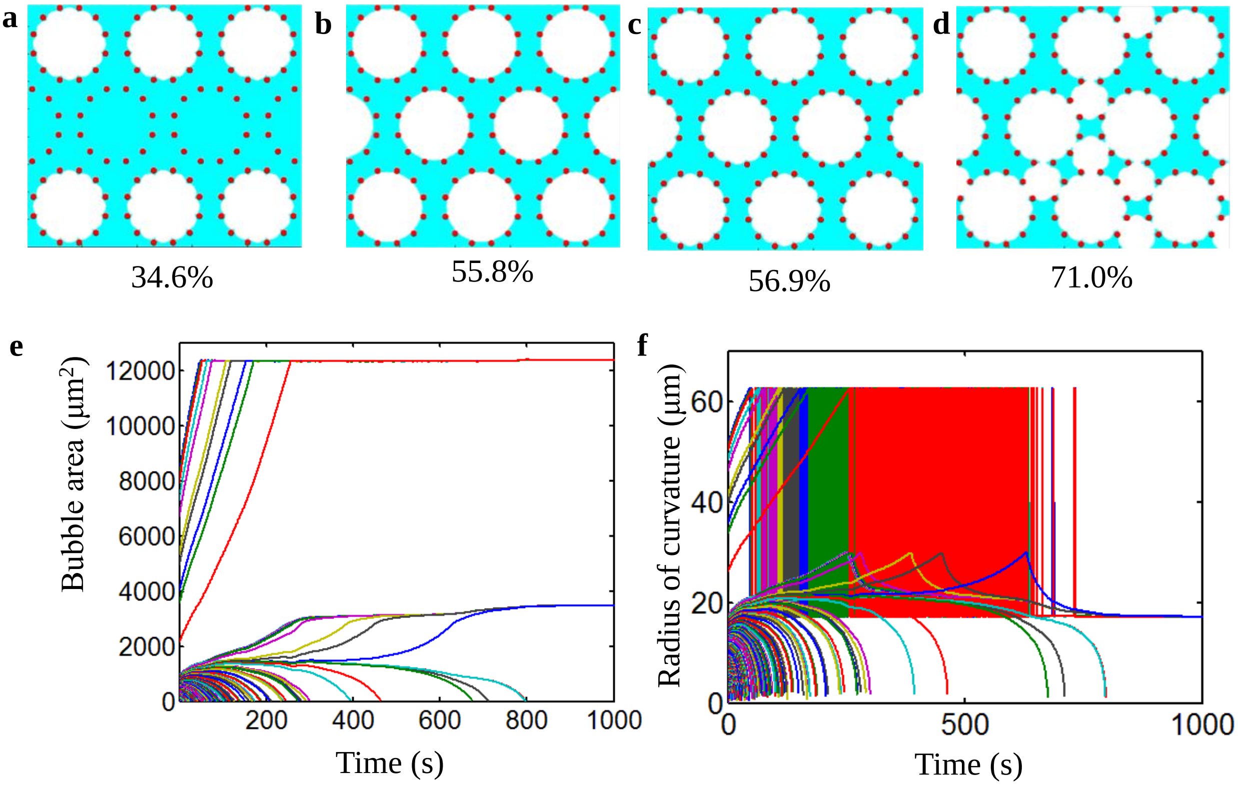

Supplementary Figure 7 │ The evolution of 2D foams with different gas volume fractions were simulated for reproducing the gather effect. (a) When the gas is insufficient to fill all the polygonal cells, the bubbles will fill the dodecagonal cells firstly. **(b-c)** As the increase of the gas volume fraction, bubbles fill all the dodecagonal cells (**b**) and deform from roundness (**c**). **(d)** When the gas is sufficient to fill all the dodecagonal cells, bubbles can also fill some of the hexagonal cells. The bubbles in hexagonal and dodecagonal cells are disconnected, and the boundaries between bubbles are not shown in the simulation. It is hard to simulate the evolution of foams with more gas volume fraction, because bubbles apart from the roundness need to be considered. **(e-f)** Area and radius of curvature variations for each bubble in the foam as a function of time in the simulation of **d**, showing that bubbles in the hexagonal and dodecagonal cells have the same surface curvature radius although they are different in area, so the system is stable and will not evolve. These results agree well with experiments shown in Supplementary Fig. 6.

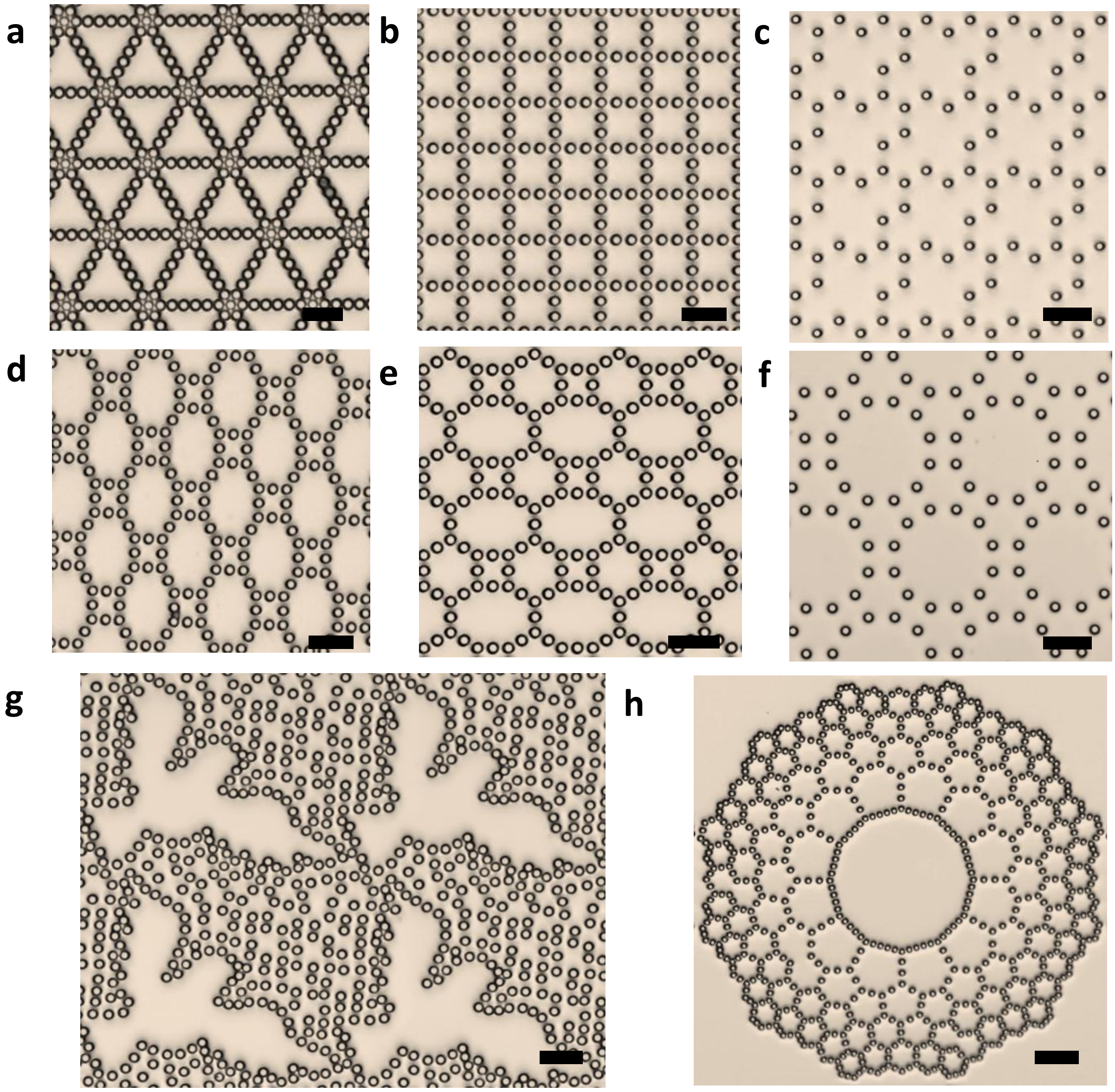

Supplementary Figure 8 │ Various pillar-structured silicon substrates were designed for preparing bubble patterns in Fig. 4. Pillars on the substrate were arranged into the patterns consisting of regular triangle (**a**), square (**b**), cross (**c**), square and regular octagon (**d**), square, regular hexagon and octagon (**e**), square, regular hexagon and dodecagon (**f**), peace-dove shape and pattern of randomly arranged dense pillars (**g**), conformal pattern of a hexagonal bubble arrays with rotational symmetry[2] (**h**). The interval of pillars in **a-c** was designed narrow enough to reduce the radius of curvature for the large bubbles to avoid them from overstepping the triangular, square or cross cells. In **d-f**, different polygonal cells were designed on the surface for exhibiting the gathering effect. For **g-h**, more complex patterns were designed for showing that various bubble patterns can be achieved based on the fundamental understanding. Scale bars, **a-g** 50 µm. **h** 100 µm.

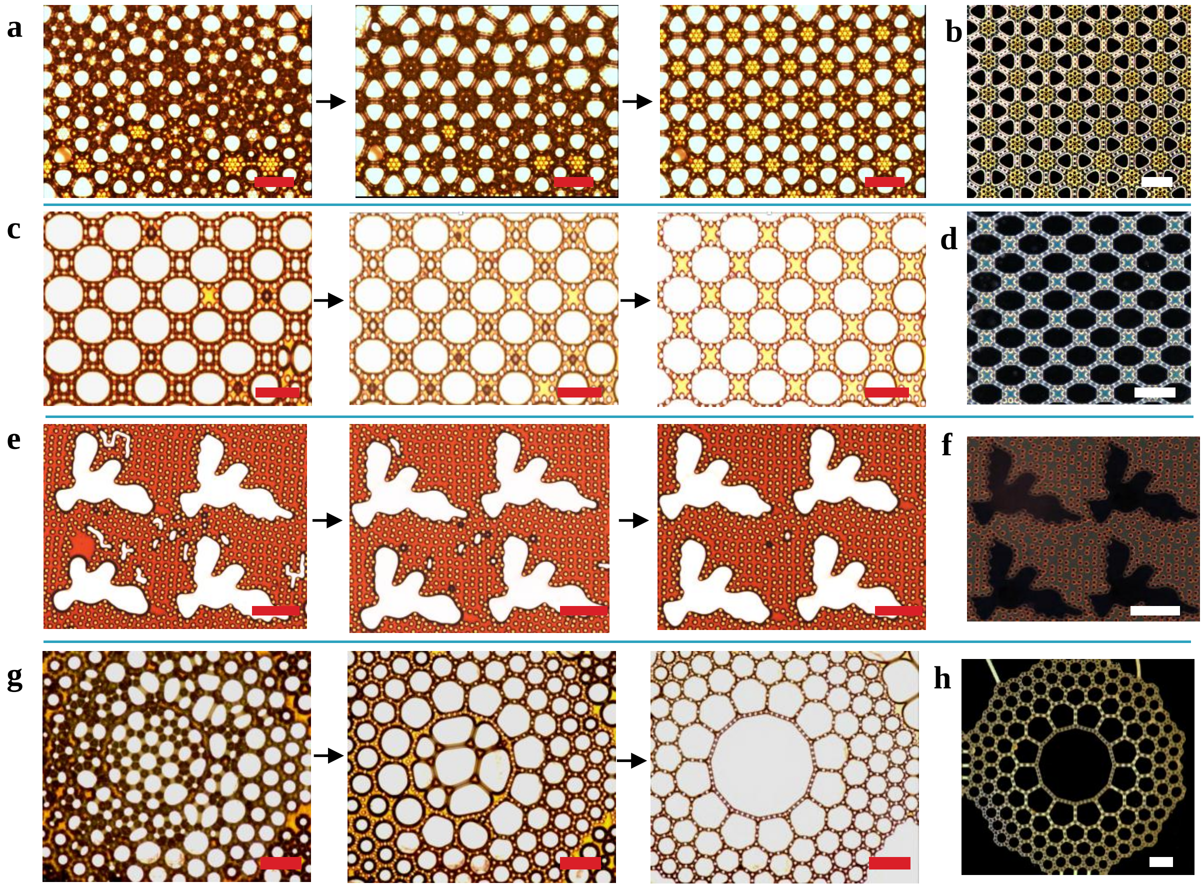

Supplementary Figure 9 │ The Forming process and corresponding dark field microscope images of bubble patterns in Fig. 4a, 4d, 4e and 4h. **(a)** By reducing the space between pillars, bubbles can be effectively trapped and shaped into triangle shape. **(c)** The octagonal cells allow bubbles having the larger radius of curvature than that of the square cells unless they are filled, so bubbles in octagonal cells can consume bubbles in square cells until all the octagonal cells were filled. **(e)** Bubbles produced both in the peace-dove-shaped cells and among the randomly arranged dense pillars, but bubbles in the peace-dove-shaped cells have the larger radius of curvature unless they fill the cells. Therefore, peace-dove-shaped cells have the gathering effect and collect surrounding bubbles of the smaller radius of curvature. **(g)** Since enough gas can fill the dodecagon cell and around large hexagonal cells at the initial state of the evolution, bubbles in the small hexagonal cells can survive from the evolution. **b, d, f, h** are dark field microscope images of experimental results of **a, c, e, g**, respectively. Scale bars, 100 μm.

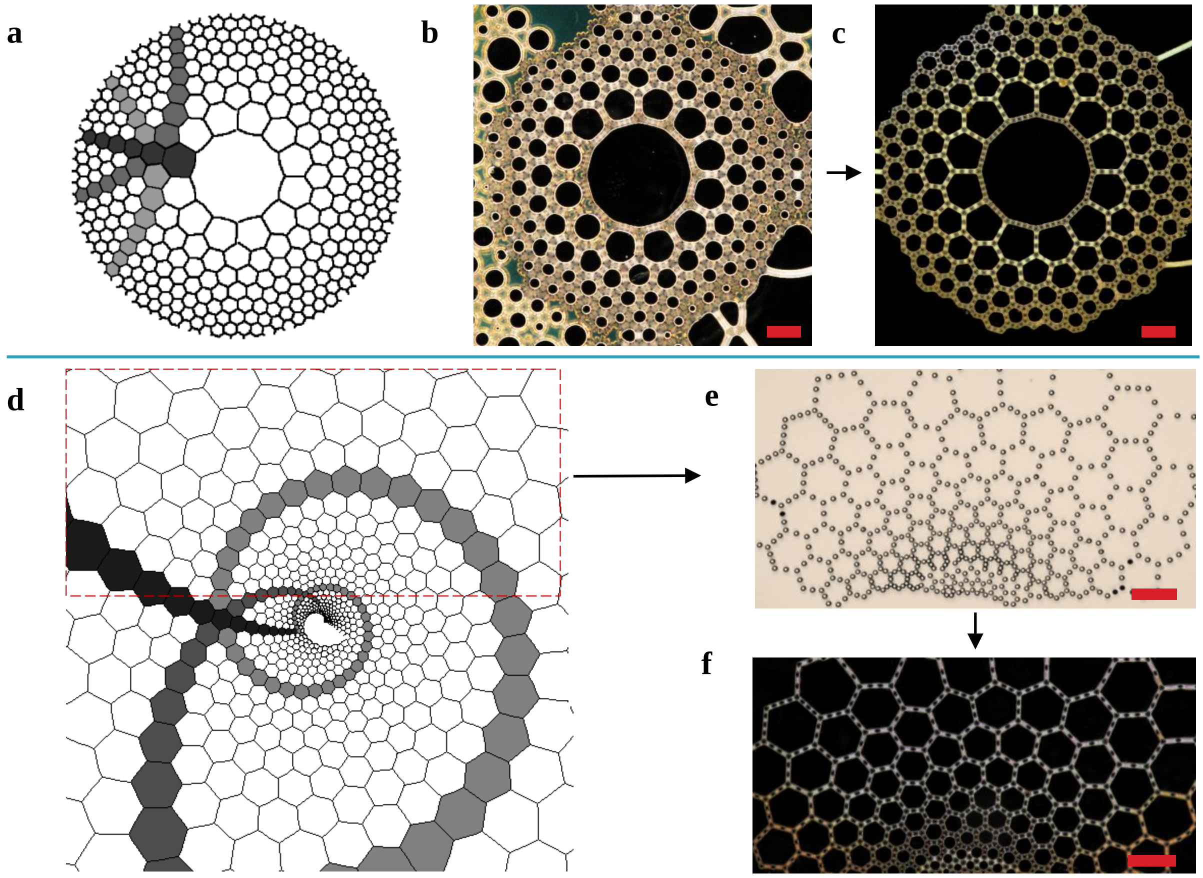

Supplementary Figure 10 | Patterns of conformal 2D foams prepared by designing the corresponding geometries of pillars arranging. **(a, d)** Numerical prediction patterns of conformal 2D foam[2] with rotational symmetry and radial symmetry, respectively. [Reproduced from ref 2 by permission from IOP Publishing, copyright 2004]. **(b-c)**, Experimental results by guiding evolution of 2D foams with the pillar-structured substrate shown in supplementary Fig. 8h. **c** is obtained from **b** through drainage due to evaporation. **(e)** Designed pillar-structured substrate for preparing part of the bubble patterns shown in **d**. **(f)** Achieved bubble patterns. All scale bars, 100 µm.

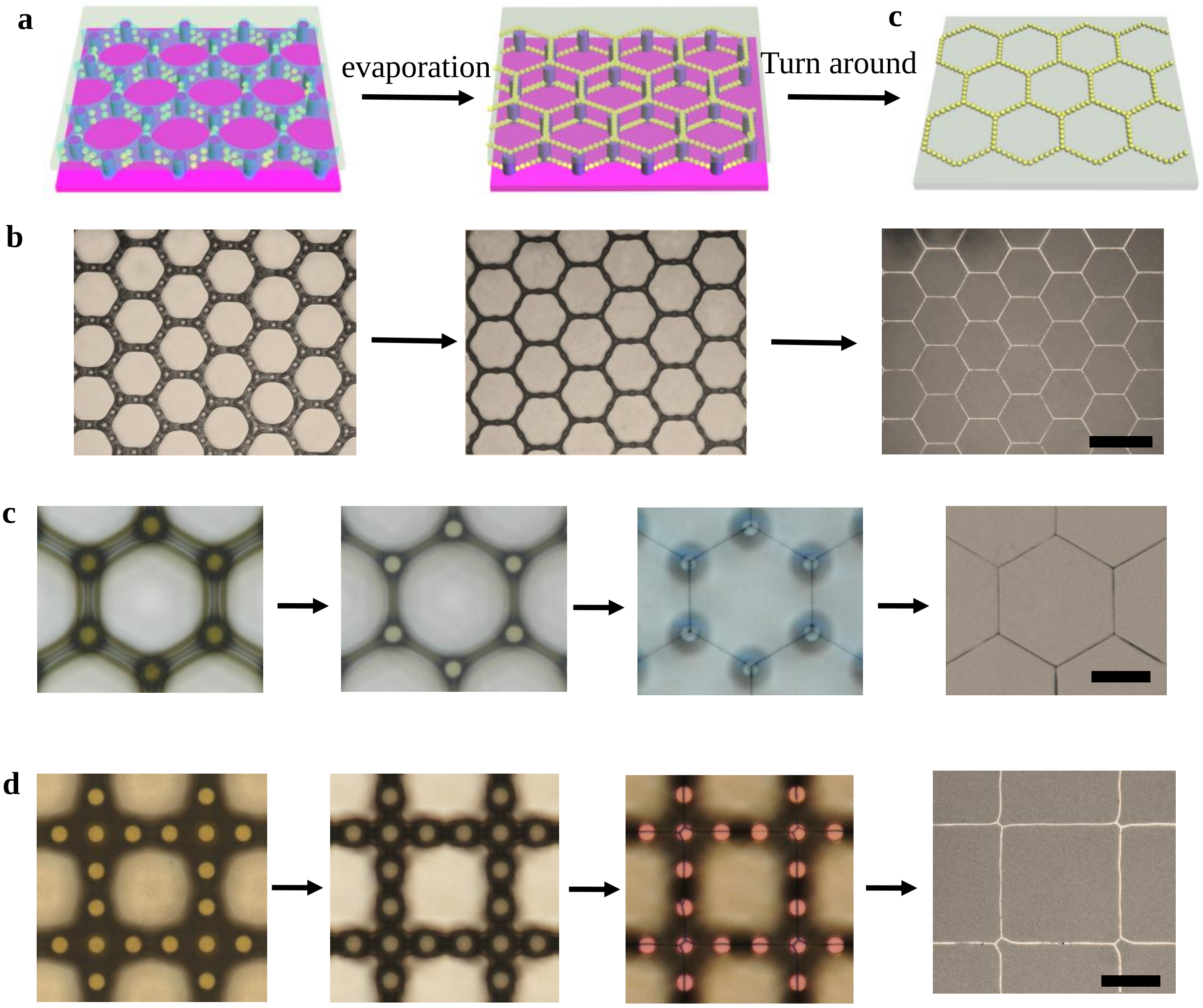

Supplementary Figure 11 │ Forming process of AgNP network with patterned bubbles as a template. (a) Schematic illustrations from a side view of the assembly process of a hexagonal network of AgNPs. **(b)** Microscope observation of the assembly process of AgNPs. **(c-d)** Magnified image shows details in the forming process of AgNP hexagonal (**c**) and square network (**d**). **(c-d)** If farther evaporation has been allowed after forming the bubble patterns, boundaries will become narrower and narrower and provide a gradually reduced confined space for AgNPs. When the liquid completely evaporates, a hexagonal network of close-packed AgNPs will form on the pillar-structured silicon substrate. Finally, removing the pillar-structured substrate, AgNP network will be remained on the glass substrate. Scale bars, **b** 100 μm, **c-d** 30 μm.

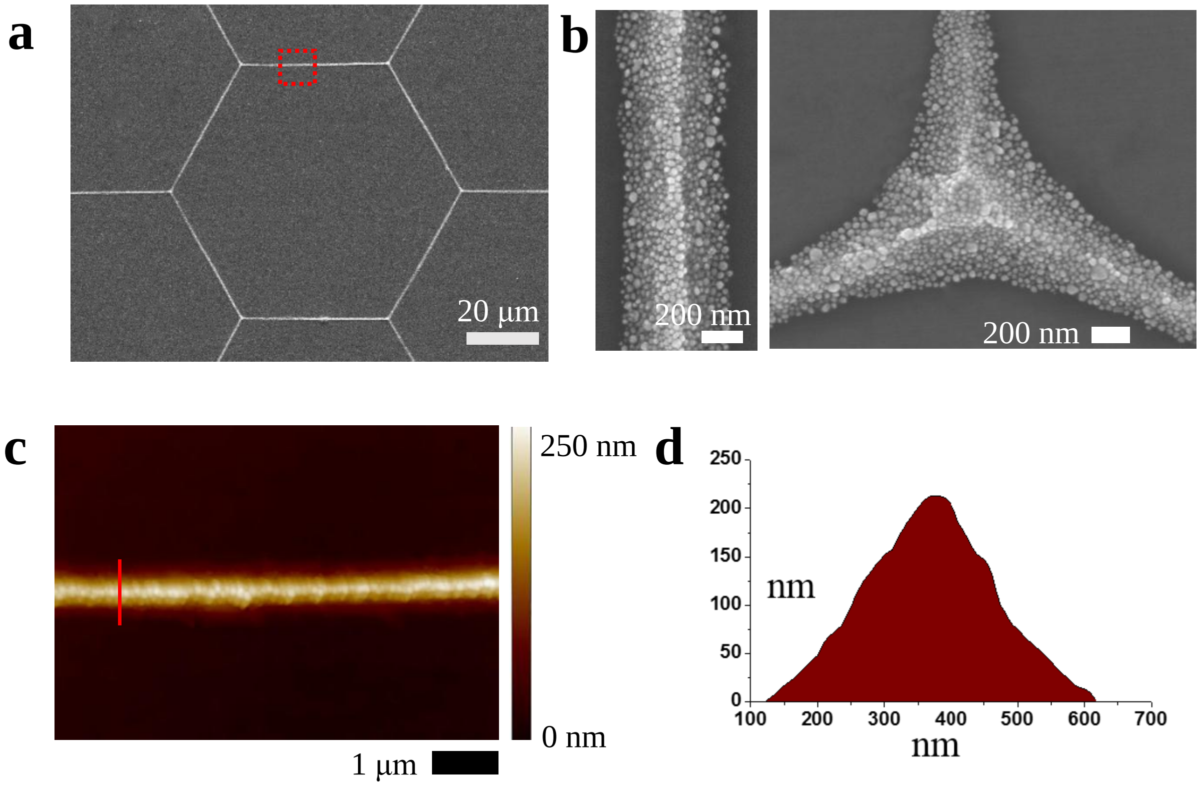

Supplementary Figure 12 │ The feature of AgNP assembly with bubbles as the template. (a) SEM image of AgNP hexagonal cell. The length and width of the hexagonal sides is 41±2 μm, 465±65 nm, respectively. **(b)** Magnified image of the line and joint of the hexagonal cell in **a**, respectively. **(c)** AFM (atomic force microscope) image at the red rectangle illustrated in **a**. **(d)** Height graph of the cross section indicated in **c** (the red line), showing a triangle-shaped aggregation of AgNPs.

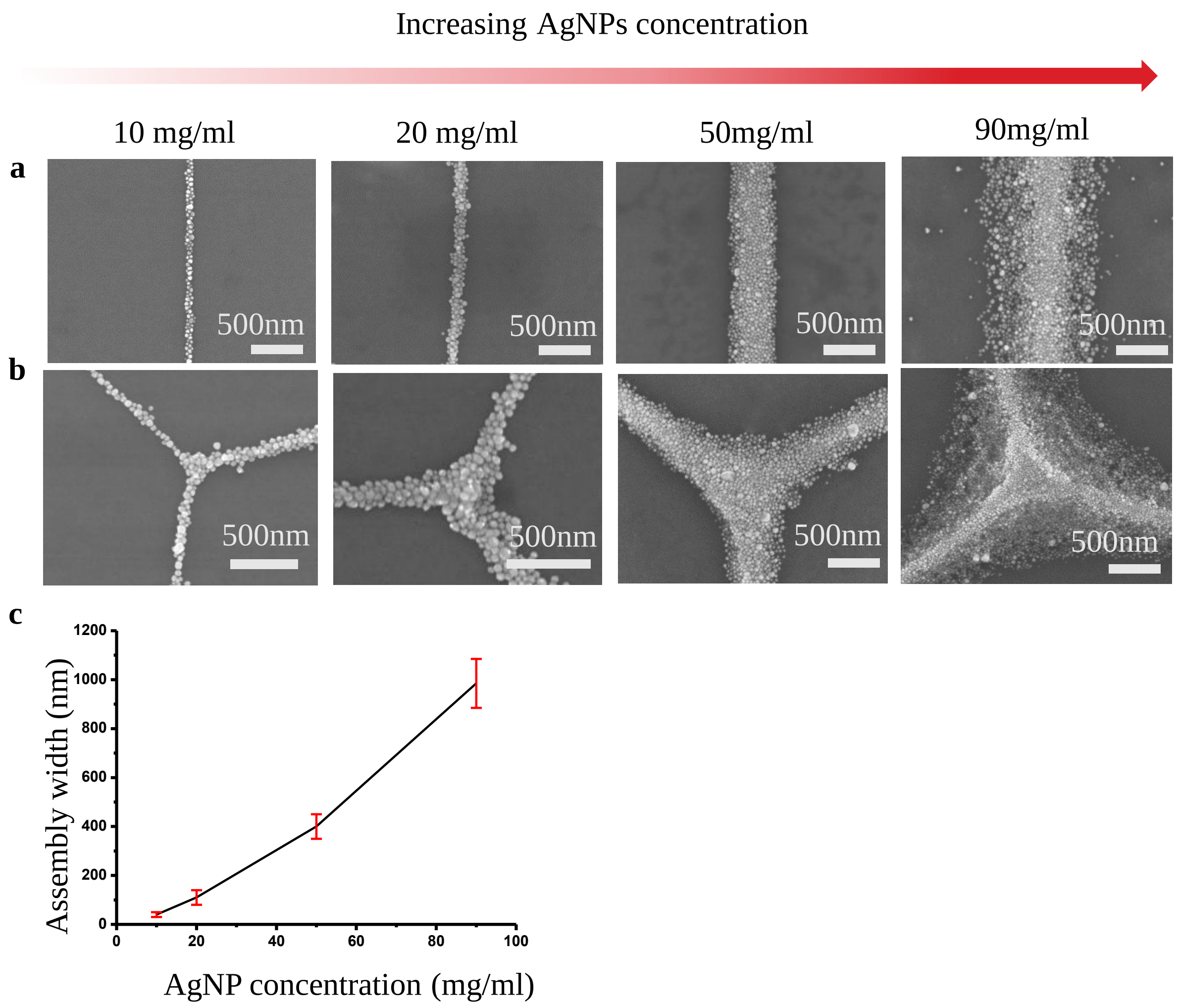

Supplementary Figure 13 │ Dependence of AgNP hexagonal network on the concentration of AgNP suspension. (a-b) SEM images of lines and joints of hexagonal network of AgNPs, respectively. **(c)** Dependence of line width of AgNP hexagonal network on the AgNP suspension. The line width can be prepared from 48±13 nm to 998±100 nm.

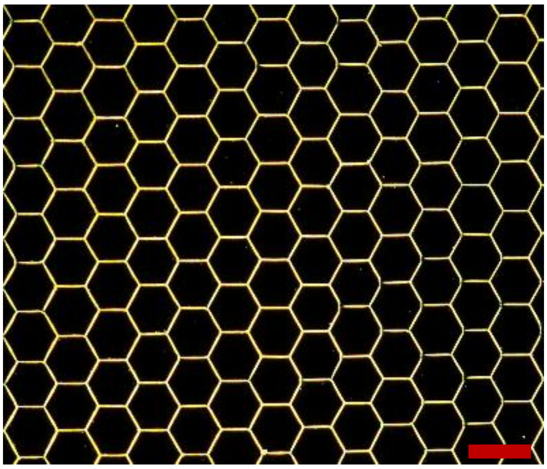

l = 40 μm

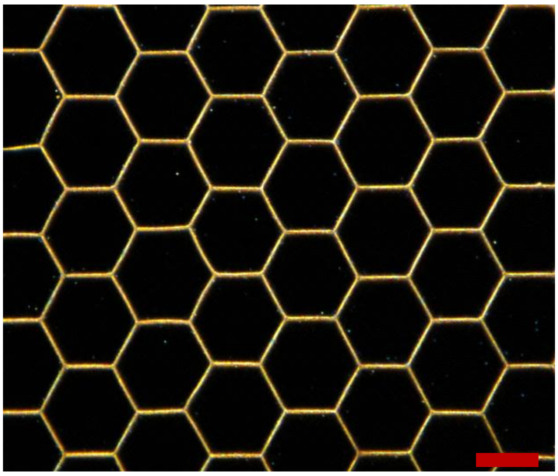

l = 70 μm

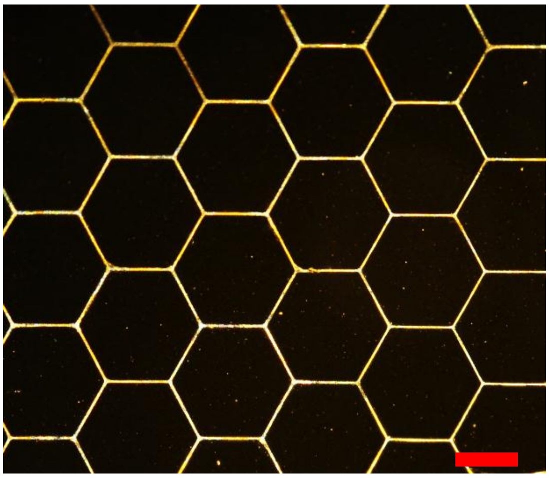

l =100 μm

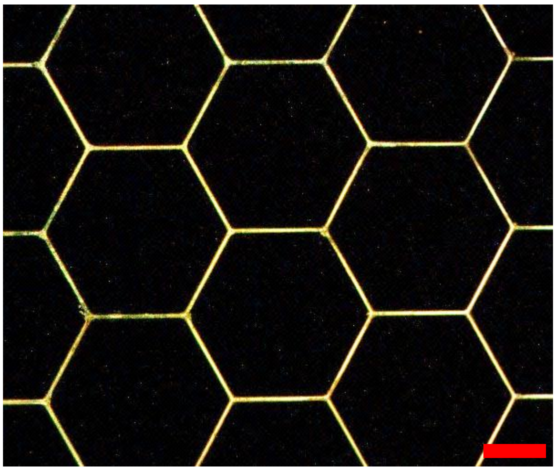

l = 160 μm

Supplementary Figure 14 | Dark field optical micrographs of the AgNP hexagonal networks with different side lengths. Scale bars, 100 μm.

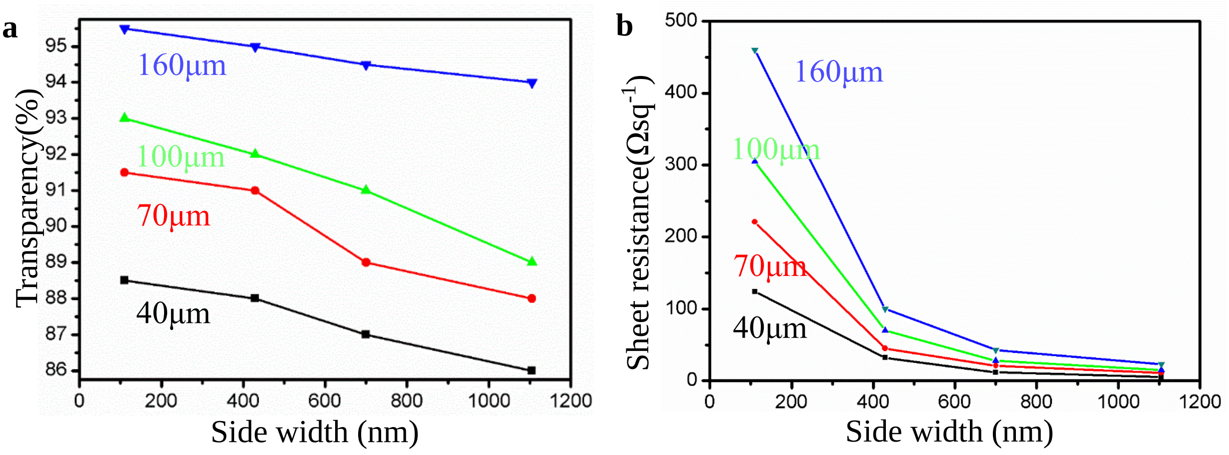

Supplementary Figure 15 │ Transparency and conductivity of the as-prepared transparent electrode. Transparency (**a**) and sheet resistance (**b**) of AgNP hexagonal network (after heat-treatment at 200℃ for 1 h) depends on side length(***a***, from 40 μm to 160 μm) and line width (from 110 nm to 1105 nm) of the hexagonal cell. The transparency is between 86% and 96%, while sheet resistance varies from 5.4 Ωsq^{-1} to 460 Ωsq^{-1}.

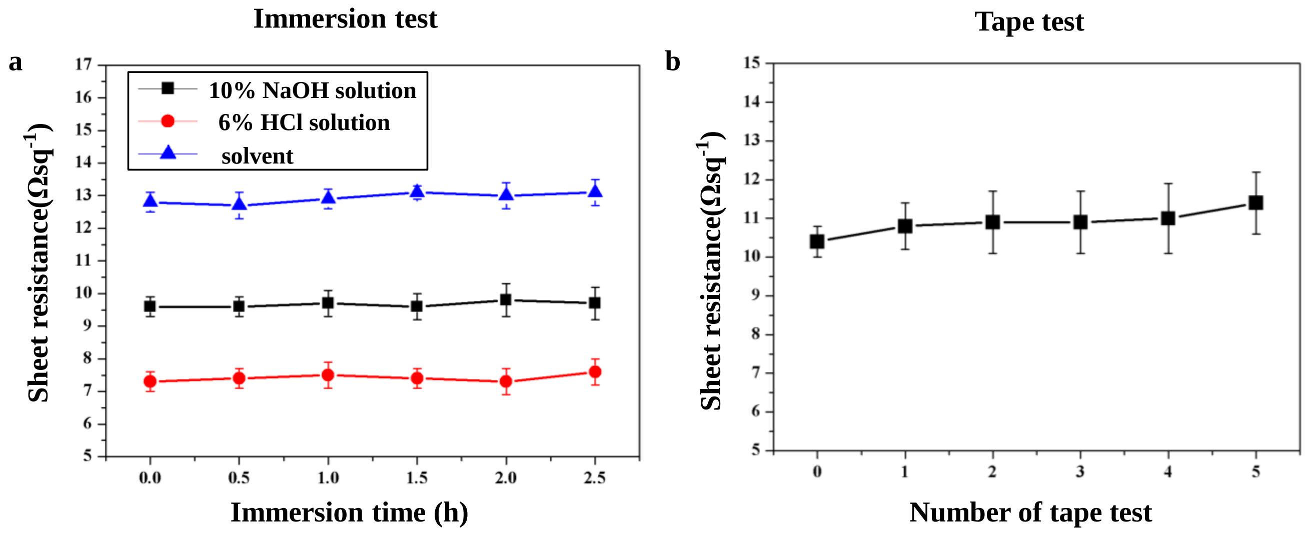

Supplementary Figure 16 | The immersion test (**a**) and tape test (**b**) of the prepared AgNP patterns. According to previously reports[4, 5], the immersion test and tape test were conducted to the AgNP patterns after sintering at 200℃ for 1 h. **(a)** Three samples were immersed in 10% water solution of NaOH at 60 ℃, 6% water solution of HCl at 25℃ and solvent composed of water, ethanol and acetone at the rate of 1:1:1 at 25℃, respectively. The conductivity was measured in every 30 minutes. The variation of conductivity is within 5% in the immersion test, showing good resistance to washing. **(b)** The tape test was carried out. The change in conductivity is within 10% after five tape tests, suggesting good performance against abrasion.

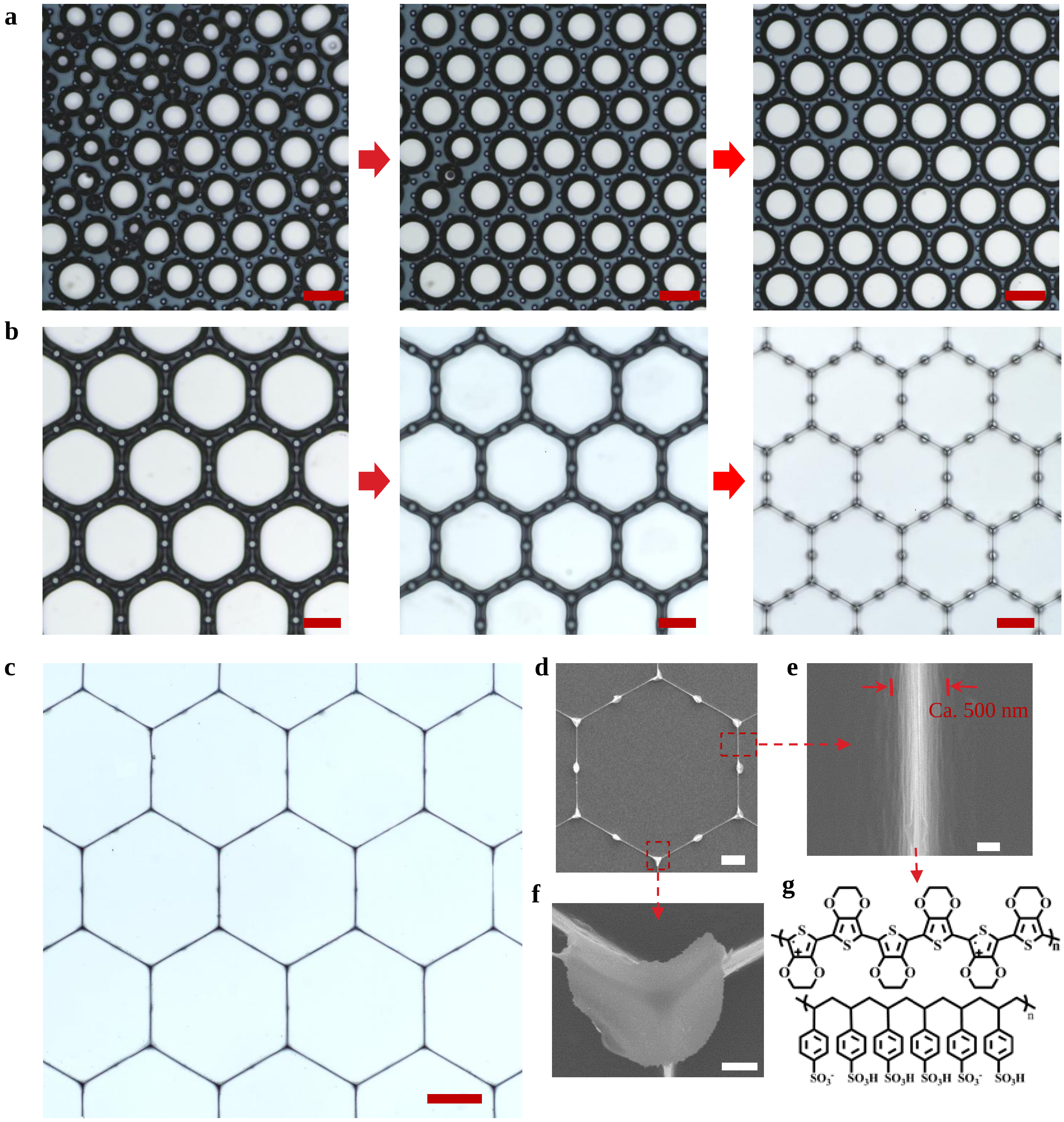

Supplementary Figure 17 | The intrinsically conductive polymer, PEDOT (poly(3,4-ethylendioxythiophene)), was assembled into hexagonal network with the patterned hydrogen bubbles as the template generated from the hydrolysis of sodium borohydride catalyzed by the PEDOT/ PSS (Poly(styrenesulfonic acid)) dispersion. (a) The evolution of 2D foams generated from hydrolysis of $NaBH_4$, which is the same with the evolution of 2D foams obtained from decomposition of urea peroxide. **(b)** The assembly of PEDOT/PSS, which is the same with the assembly of AgNPs. **(c)** Bright field microscope images of obtained PEDOT/PSS network. **(d-f)** SEM images of PEDOT/PSS network. **(g)** The molecular structural formulas of PEDOT (top) and PSS (bottom). The hydrogen ions dissociated from PSS in water can catalyze the hydrolysis of $NaBH_4$ to produced hydrogen bubbles. Scale bars, **a** 100 μm, **b-c** 50 μm, **d** 20 um, **e** 200 nm, **f** 500 nm.

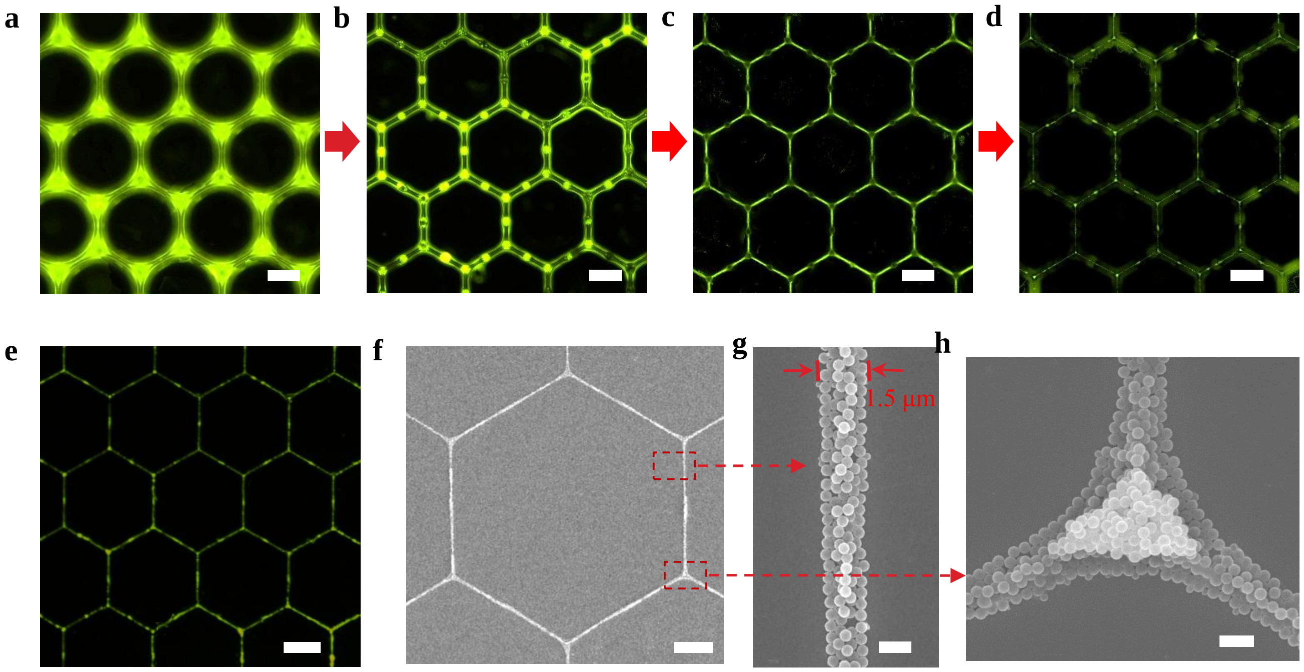

Supplementary Figure 18 | Polystyrene (PS) microspheres (average size of 450 nm and with fluorescent molecules modified on the surface) were assembled with the patterned hydrogen bubbles as the template generated from hydrolysis of $NaBH_4$ catalyzed by H^+. (a-d) Fluorescent micrographs showing the assembly process of PS microspheres, which is the same with the assembly of AgNPs with patterned oxygen bubbles as the templates. **(e)** Fluorescent micrographs of prepared PS microsphere hexagonal network. **(f-h)** SEM images of obtained PS microsphere network. Scale bars, **a-e** 50 μm, **f** 20 μm, **g-h** 1 μm.

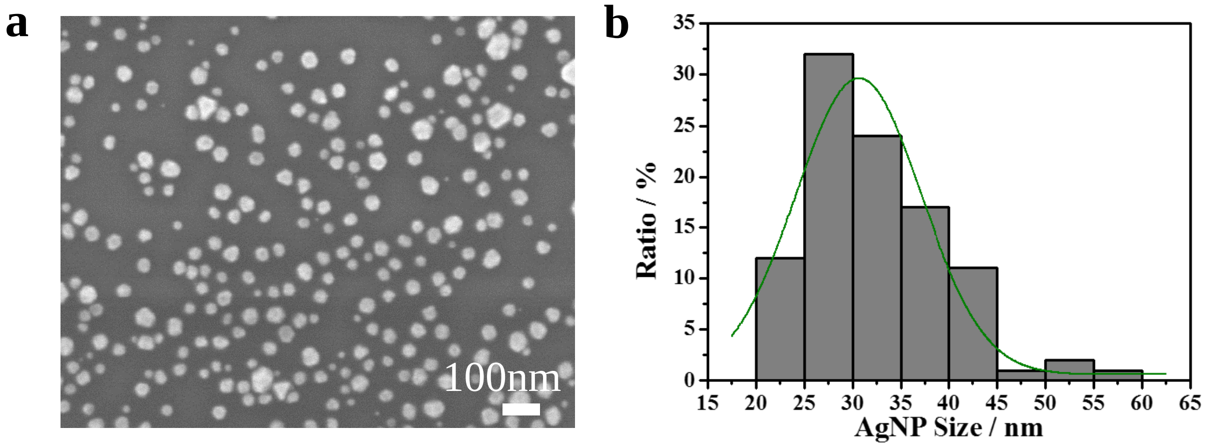

Supplementary Figure 19 │ Preparation of silver nanoparticles (AgNPs). (a) SEM image of AgNPs upon a pre-clean flat silicon wafer. **(b)** Statistical size distribution of AgNPs from 100 particles in the SEM observation, showing their average size is 33 ± 2 nm.

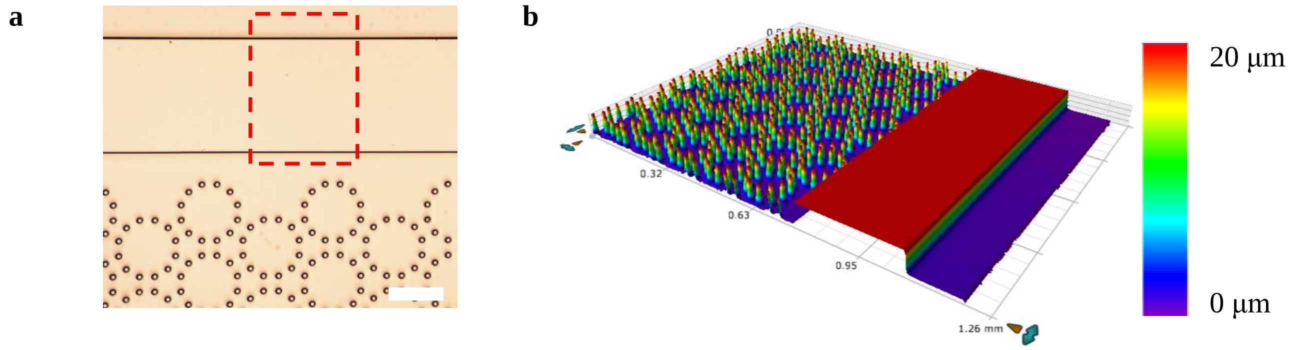

Supplementary Figure 20 | Designed pillar-structured silicon substrate to avoid evaporation from side edges during the evolution. The wall (the dashed red rectangle in **a**) surrounding the pillars has the same height with pillars (**b**), which avoids evaporating from the side edges. **(a)** Optical micrographs from top view of the patterned substrate. **(b)** Surface profiles of the patterned surface. Scale bar, **a** 100 μm.

Supplementary Note 1

Effect of pillars on radius of curvature for the growing bubble in square, hexagonal and dodecagonal cells.

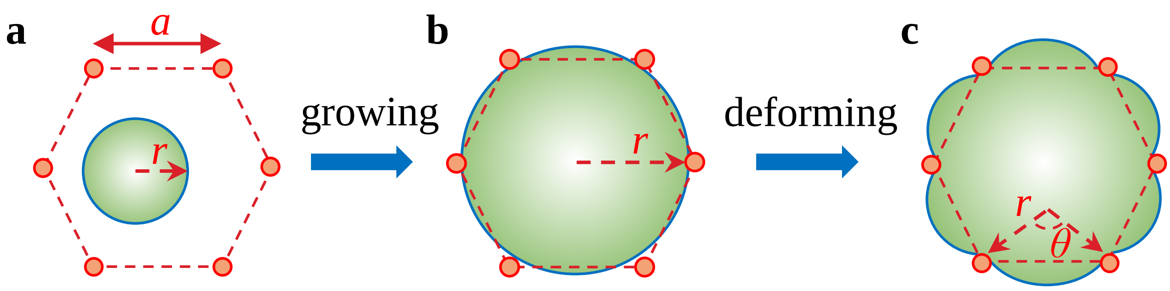

Supplementary Scheme 1 | Schematic illustrations of a bubble growing and deforming in a hexagonal cell consisting of pillars. *a* denotes side length of the hexagonal cell (namely, the distance between adjacent silicon pillars), and θ is the central angle of each arc in **c**, which satisfying, $\sin(\theta/2) = a/2r$.

Here, we take the hexagonal cell as an example to study the effect of pillars on radius of curvature of the growing bubble. Square and dodecagonal cells are similar to this. For bubbles between two substrates, we can treat surface area as volume because $V = Ah$, where V, A are volume and projected area of a bubble from the top view, respectively, h is thickness of the confined 2D space and approximates to the height of the pillar on the substrate. L is the perimeter of the boundaries of projected area bubbles from top view. The radius of pillars can be negligible for simplicity, and pillars can be taken as discrete points for interacting with bubbles. As Supplementary Scheme 1 shows, if A is smaller than or equal to πa^2, $A = \pi r^2$, $L = 2\pi r$. But if A is larger than πa^2, bubble deforms from roundness and r decreases, so A is larger than πr^2. The relation between V and r can be obtained as follows,

$$A = 6\left(\frac{1}{2}\theta r^2 - \frac{a^2}{4\tan\frac{\theta}{2}}\right) + \frac{3\sqrt{3}}{2}a^2$$ and $L = 6\theta r$, where $\sin(\theta/2) = a/2r$.

Similarly, surface curvature radius of growing bubble in the square, dodecagonal cells can be deduced, and the results are summarized below,

$$A = f(r),\quad L = f(r) \quad \begin{cases} A \in [0, \dfrac{\pi a^2}{4\sin^2(\pi/n)}] & L = 2\pi r \qquad A = \pi r^2 \\ A \in [\dfrac{\pi a^2}{4\sin^2(\pi/n)}, \infty] & L = n\theta r \qquad A = (\dfrac{a^2}{4\tan(\pi/n)} + \dfrac{1}{2}\theta r^2 - \dfrac{a^2}{4\tan(\theta/2)}) \times n \qquad \sin(\theta/2) = a/2r \end{cases}$$

Where n = 4, 6, 12 and denotes bubbles growing in square, hexagonal and dodecagonal cells. For equation above, it can be deduced that

$$r = \frac{a}{2\sin(\theta/2)} \geq \frac{a}{2}$$

Therefore, the minimum curvature radius of a bubble that pillars can reduce to is $a/2$ (when $\theta = \pi$). Surrounding bubbles with radii less than $a/2$ can be adsorbed by this larger deformed bubble, which assists to form defects. So the pillar interval is very important for guiding evolution of 2D gas-liquid foams into patterns as desired.

Supplementary Note 2

Governing equation for 2D gas-foams evolution (focusing on the foams where most bubbles can maintain the roundness)

1. The pressure in a bubble

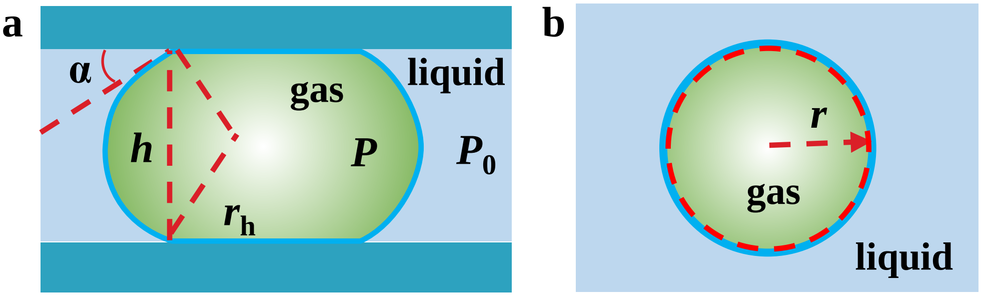

Supplementary Scheme 2 | Schematic illustrations of a bubble between two substrates from the side view (**a**) and top view (**b**). α denotes the contact angle of the cover plate, and h is the distance between two substrates. r_h and r are principal radii of gas-liquid interface of the bubble, P is the pressure in the bubble, and P_0 is the external pressure. The dashed red circle in b indicates the interface where gas exchange happens.

Bubbles in 2D space can be illustrated as Supplementary Scheme 2. According to Laplace-Young equation, the pressure in a bubble is determined by its principal radii (r_h, r), external pressure (P_0) and

surface tension (σ), so $P = P_0 + \sigma/r_h + \sigma/r$, where $r_h = h/(2\cos\alpha)$, and r_h can be assumed constant (because h and α are constant) for different bubbles. So for 2D bubbles with radius of r_1 and r_2, the difference in pressure, $\Delta P = \sigma(1/r_1 - 1/r_2)$.

2. Governing equation for 2D gas-liquid foam evolution

According to Lemlich's theory[3], the concentration of gas in liquid can be considered as being equivalent to a gas pressure in the liquid through Henry's law, and this equivalent gas pressure can be viewed as a fictitious 2D bubble with a radius of ρ, so the pressure difference between a bubble of any size and the liquid satisfying $\Delta P = \sigma(1/\rho - 1/r)$. From general rate equation, $Q_m = -JA_s\Delta P = dm/dt$, Where Q_m is the molar rate of gas transfer from a bubble to the liquid, J, the effective permeability to the transfer, A_s, the gas-liquid interface where gas exchange takes place, and m, the moles of gas in the bubble. Supplementary Scheme 2 shows, $A_s \approx Lh$, where L has been defined in Supplementary Note 1. So $dm/dt = Jh\sigma(L/\rho - L/r)$. Using conservation of gaseous moles throughout the foam as a whole, namely, $\sum_{i=1}^{n} Q_{mi} = 0$ we can get that $\rho = \sum_{i=1}^{n} L_i / \sum_{i=1}^{n} \frac{L_i}{r_i}$.

By assuming that gas in the bubble is an ideal gas, so $PV = mRT$, where P is the pressure in the bubble, R is the ideal gas constant , T is the absolute temperature, V is the volume of the bubble, and approximately $V = Ah$, A is the projected area of the bubble from the top view. For bubbles with radius more than 10 μm, $P_0 >> \sigma/r + \sigma/h$, and P can be assumed to be equal to P_0, so

$$\frac{dA}{dt} = \frac{RTJ\sigma}{P_0}\left(\frac{L}{\dfrac{\sum_{i=1}^{n} L_i}{\sum_{i=1}^{n} \frac{L_i}{r_i}}} - \frac{L}{r}\right)$$

For simplification, if assuming $K = RTJ\sigma/P_0$, and retaining ρ in the equation, then

$$\frac{dA}{dt} = KL\left(\frac{1}{\rho} - \frac{1}{r}\right)$$

According to Lemlich's theory[3], ρ is instantaneous mean radius of the foams, and will gradually increase during the evolution. At any time, bubbles with radius of curvature, r, larger than ρ will grow, while bubbles with r less than ρ will shrink. If $a/2$ is less than ρ, the deformed larger bubbles will shrink once their radii of curvature reach the minimum radius of curvature, $a/2$. So the reverse Ostwald ripening will always happen. Since ρ gradually increases during the evolution, the minimum

ρ is at the initial state of the evolution (denoted as ρ_0). Therefore keeping $a/2 < \rho_0$ can effectively forbid the defect formation.

Supplementary Note 3

1. **The design of pillar interval for trapping growing bubble into any polygonal cell consisting of discrete pillars.**

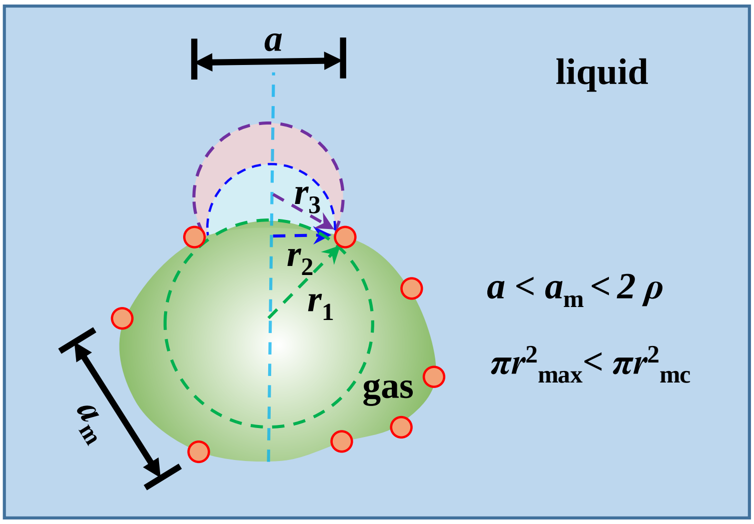

Supplementary Scheme 3 | Schematic illustrations for design of pillar intervals when trapping a growing bubble in a polygonal cell. The r_1, r_2, r_3 are curvature radii of the meniscus when the meniscus passes through two adjacent pillars. a_m donates the maximal interval of adjacent pillars. ρ, r_{max} donate the instantaneous mean radius, the maximal radius of bubble of the foams at the first of the evolution, respectively. πr^2_{mc} is the allowed maximal circular size of the bubbles in the polygonal cell. The r_{mc} is the corresponding radius of the maximal circle, and means the allowed maximal radius of curvature for the bubbles in the cell.

As shown in Supplementary Scheme 3, when a growing bubble (the green bubble shown in the scheme) interacts with pillars and deforms into several meniscuses, each meniscus has a reduced radius of curvature and its minimum is half of the corresponding pillar interval ($a/2$). For example, as shown in the scheme, $r_2 = a/2$. For polygonal cell with different pillar intervals, making sure that $a_m < 2\rho$ can avoid all the meniscuses passing through the gap of pillars, thus trapping the growing bubble. Furthermore, for preparing perfect bubble arrays, the maximal circle size of the polygonal cell should

be larger than that of the maximal bubble at the first of the evolution. So the pillar intervals should satisfy $a_m < 2\rho$, and the designed polygonal cell satisfies $\pi r^2_{max} < \pi r^2_{mc}$.

2. Explaining the gathering effect in any polygons.

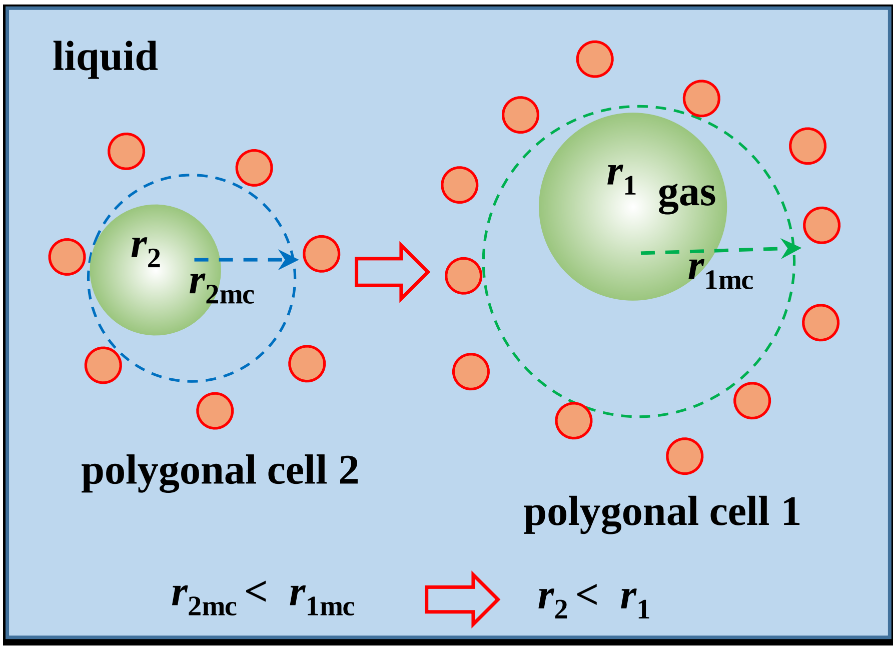

Supplementary Scheme 4 │ Schematic illustrations for explaining the gathering effect in any polygons. Polygonal cell 1 and polygonal cell 2 are any two polygonal cells where bubbles can have the allowed maximal curvature radius of bubble r_{1mc}, r_{2mc}, respectively, and $r_{2mc} < r_{1mc}$. r_1 and r_2 are curvature radius of bubbles in the two polygonal cells.

Assuming that pillars on the silicon substrate were arranged into various polygonal cells. Polygonal cell 1 and polygonal cell 2 are any two polygonal cells. Assuming that $r_{2mc} < r_{1mc}$ and gas distributes uniformly in the confined 2D space, more gas will distribute in polygonal cell 1 than polygonal cell 2 because it has a larger area. With evolution, bubbles in a polygonal cell will merge into a large bubble, as r_1, r_2 shown in the scheme. So $r_2 < r_1$ because more gas has distributed in polygonal cell 1. The bubble in polygonal cell 1 will consume that of polygonal cell 2 owing to the unbalance pressure until polygonal cell 1 was filled. Therefore, when the total gas is not enough and distributes uniformly, the bubbles will evolve into domains which allow them having a larger radius of curvature until filling these domains.

Supplementary Reference

1. Xin, Z. *et al.* Continuous microwire patterns dominated by controllable rupture of liquid films. *Small* **9**, 722-726 (2013).
2. Drenckhan, W., Weaire, D. & Cox, S. J. The demonstration of conformal maps with two-dimensional foams. *Eur. J. Phys.* **25**, 429-438 (2004).
3. Lemlich, R. Prediction of Changes in Bubble Size Distribution Due to Interbubble Gas Diffusion in Foam. *Ind. Eng. Chem. Fundam.* **17**, 89-93 (1978).
4. Jin Y, *et al.* Annealing-free and strongly adhesive silver nanowire networks with long-term reliability by introduction of a nonconductive and biocompatible polymer binder. *Nanoscale*. **6**, 4812-4818 (2014).
5. Layani M, Magdassi S. Flexible transparent conductive coatings by combining self-assembly with sintering of silver nanoparticles performed at room temperature. *J. Mater. Chem*. **21**, 15378-15382 (2011).
